# Long-term orange juice consumption is associated with low LDL-cholesterol and apolipoprotein B in normal and moderately hypercholesterolemic subjects

**DOI:** 10.1186/1476-511X-12-119

**Published:** 2013-08-06

**Authors:** Nancy P Aptekmann, Thais B Cesar

**Affiliations:** 1 Department of Food and Nutrition, School of Pharmaceutical Sciences, UNESP - Univ Estadual Paulista, Araraquara, SP1 14801-902, Brazil

**Keywords:** Orange juice, LDL-cholesterol, Apo B, Homocysteine, Humans, Suco de laranja LDL-colesterol, Apo B, Homocisteína, Humanos

## Abstract

**Background:**

This study investigated the hypothesis that long-term orange juice consumption (≥ 12 months) was associated with low risk factors for cardiovascular disease in adult men and women with normal and moderately high cholesterol blood levels.

**Methods:**

The sample consisted of 103 men (18–66 y) and 26 women (18–65 y); all were employees of an orange juice factory with daily access to free orange juice. The results showed that 41% of the individuals consumed 2 cups (480 mL) of orange juice per day for at least twelve months, while 59% of the volunteers are non-consumers of orange juice.

**Results:**

Orange juice consumers with normal serum lipid levels had significantly lower total cholesterol (−11%, p <0.001), LDL-cholesterol (−18%, p < 0.001), apolipoprotein B (apo B) (−12%, p < 0.01) and LDL/HDL ratio (−12%, p < 0.04) in comparison to non-consumers, as did the consumers with moderate hypercholesterolemia: lower total cholesterol (−5%, p <0.02), LDL-cholesterol (−12%, p <0.03), apolipoprotein B (−12%, p <0.01) and LDL/HDL ratio (−16%, p <0.05) in comparison the non-consumers counterparts. Serum levels of homocysteine, HDL- cholesterol and apolipoprotein A-1, body composition and the dietary intake of food energy and macronutrients did not differ among orange juice consumers and non-consumers, but vitamin C and folate intake was higher in orange juice consumers.

**Conclusion:**

Long-term orange juice consumers had lower levels of total cholesterol, LDL-cholesterol, apo B and LDL/HDL ratio and an improvement of folate and vitamin C in their diet.

## Abstract (de)

**Resumo:**

**Introdução**

O presente estudo investigou a hipótese de que o consumo de suco de laranja a longo prazo (≥ 12 meses) foi associado com menor fator de risco para doença cardiovascular em homens e mulheres adultos com nível normal e moderadamente alto de colesterol no sangue.

**Métodos**

A amostra foi composta por 103 homens (18–66 anos) e 26 mulheres (18–65 anos) todos eram funcionários de uma fábrica de suco de laranja com acesso diário e gratuito ao suco de laranja. Os resultados mostraram que 41% dos indivíduos consumiram dois copos (480 mL) de suco de laranja por dia durante pelo menos 12 meses, enquanto 59% dos voluntários não eram consumidores de suco de laranja.

Os consumidores de suco de laranja com níveis normais de lipídeos séricos apresentaram menor teor de colesterol total (−11% p < 0.001), LDL-colesterol (−18%, p <0.001), apolipoproteína B (apo B) (−12%, p <0.01) e LDL/HDL (−12%, p <0.04) em comparação com os não-consumidores; assim como os consumidores com hipercolesterolemia moderada (−5% p <0.02), LDL-colesterol (−12%, p <0.03), apolipoproteína B (−12%, p <0.01) e LDL/HDL (−16%, p <0.05) em comparação aos seus pares de não-consumidores. Os níveis séricos de homocisteína, HDL-colesterol e apolipoproteína A-1, a composição corporal e a ingestão de energia e de macronutrientes da dieta não diferiram entre os consumidores de suco de laranja e não-consumidores, mas a vitamina C e o folato estavam mais elevados na dieta nos consumidores de suco de laranja.

**Conclusão**

Os consumidores de suco de laranja de longo prazo apresentaram menores níveis de colesterol total, LDL-colesterol, apo B e razão LDL/HDL e uma melhoria nos níveis de folato e vitamina C nas suas dietas.

## Background

Consumption of fruits and vegetables is associated with a lower risk of coronary artery disease and this effect is attributed mainly to the functional and antioxidant actions of vitamins and phytochemicals, such as phenolic compounds and flavonoids [1-5]. In developed countries, foods such as wine, grapes and tea provide the greatest proportion of dietary flavonoids, followed by citrus fruits and orange juice [6]. Non-starchy vegetables and fruits, such as lettuce, tomatoes, onions and citrus provide 60 to 106 mg of flavonoids per day to the Brazilian diet, and oranges and fresh squeezed orange juice are responsible for more than 70% of the total flavonoid intake [7].

Orange juice contains considerable amounts of flavonoids, mainly hesperidin, and its content is related to juice processing conditions. Experimental studies have shown that hesperidin has anti-inflammatory, antiallergic, hypolipidemic and anticarcinogenic properties [7-12]. These flavonoids help to lower serum levels of LDL-cholesterol [13-15] and apo B [16,17], and can reduce triglycerides [16] and increase HDL-cholesterol [18], reducing risk of coronary artery disease [10,19,20]. The antioxidant effect of vitamin C is vasoprotective [20,21] and even small amounts of folate have been associated with lower homocysteine levels and less thromboembolic events [22].

Dyslipidemias are the main risk factors for the development of atherosclerosis, followed by visceral fat and obesity, which are related to higher rates of morbidity and mortality from coronary artery disease [23]. Lower total and LDL-cholesterol levels have been associated with decreased risk of coronary artery disease and related conditions, and thus vegetable-based diets are recommended for the treatment of dyslipidemias, not only because of low saturated fat and cholesterol, but also because of the significant amounts of micronutrients and bioactive compounds [24-26].

The actions of citrus flavonoids have been investigated in clinical and experimental studies, but most studies used high doses of isolated forms of these compounds, such as hesperidin or naringin [5,16,27]. Clinical studies with normolipidemic and hypercholesterolemic subjects committed to a regular consumption of orange juice have shown decreased LDL-cholesterol levels [13,14] and increased HDL-cholesterol levels [18]. However, these effects were not yet investigated in subjects with non-controlled long-term daily orange juice consumption.

The present cross-sectional study investigated the hypothesis that a daily long-term consumption of orange juice is associated with an improvement of lipid profile and as such, helps to lower the risk factors of cardiovascular disease in normolipidemic and moderately hypercholesterolemic adults. To test this hypothesis we compared biochemical, anthropometric and dietary parameters of regular consumers and non-consumers of commercially processed orange juice. To avoid external influences, none of the subjects were under cholesterol-lowering medication.

## Results

All women (n = 36) and 62% of the men (n = 64) had normal serum cholesterol levels (total cholesterol < 6.2 mmol/L) and 38% of the men (n = 39) had moderately high serum cholesterol levels (total cholesterol ≥ 6.2 mmol/L). The individuals were divided by total serum cholesterol and gender to study the anthropometric and dietary variables and only by total serum cholesterol to study the biochemical variables.

Nutritional assessment of the men and women with normal and men with high total cholesterol levels, grouped according to orange juice consumption, revealed no differences among the groups regarding daily intake of food energy, protein, carbohydrate, dietary fiber, total fruit, total fat, saturated fat, cholesterol and potassium (p > 0.05) (Table 1). However, vitamin C and folate intakes, calculated from the 24-hour recall, were greater in individuals who consumed orange juice regularly than in those who did not, regardless of group (p ≤ 0.05) (Table 1). The mean orange juice intake of regular consumers, regardless of gender, was 480 ± 158 mL/d, ranging from 240 to 720 mL/d. In women, the mean orange juice intake was 390 ± 149 mL/d, representing 7.9% of the total dietary energy and 14.6% of the total dietary carbohydrate. In men, regardless of cholesterol level, the mean orange juice intake was 476 ± 158 mL/d, representing 7.8% of the total dietary energy and 15.3% of the total dietary carbohydrate. Therefore, the percentage energy and carbohydrate intakes from orange juice for men and women were very similar (Table 1).

**Table 1:** Nutrient intake of orange juice consumers and non-consumers according to gender and serum cholesterol level

	**Normolipidemic**^**#**^				**Hypercholesterolemic**^**##**^	
**Subjects**	**Women**		**Men**		**Men**	
*n*	18	8	38	26	23	16
Orange juice	Non-consumers	Consumers	Non-consumers	Consumers	Non-consumers	Consumers
mL/d	0	390 ± 149	0	466 ± 163	0	486 ± 145
MJ/d	0	0.74 ±0.28	0	0.89 ±0.30	0	0.92 ± 0.27
Total Energy(MJ/d)	8.01 ± 2.02	9.44 ±2.25	11.5 ± 5.04	11.6 ±2.51	12.0 ± 2.88	11.8 ± 2.0
Protein (g/d)	83.1 ± 28.4	112 ± 38.7	117 ± 20.1	125 ± 28.4	132 ± 33.6	143 ± 25.8
Carbohydrate (g/d)	233 ± 97.3	307 ± 104	327 ± 111	346 ± 113	382 ± 123	367 ± 72.3
Dietary fiber (g/d)	12.0 ± 6.8	14.7 ± 10.0	23.5 ± 12.2	25.2 ± 14.0	27.0 ± 14.0	23.4 ± 11.6
Total fruits (g/d)	93 ± 95	89 ± 114	138 ± 184	94 ± 149	157 ± 197	125 ± 138
Total fat (g/d)	61.5 ± 22.1	62.2 ± 10.6	94.5 ± 32.4	98.6 ± 28.2	90.3 ± 37.8	90.6 ± 27.4
Saturated fats (g/d)	19.3 ± 9.37	18.9 ± 4.79	30.1 ± 12.35	31.3 ± 10.3	27.8 ± 10.2	29.3 ± 12.8
Cholesterol (mg/d)	211 ± 137	317 ± 62.4	316 ± 113	345 ± 109	317 ± 116	345 ± 123
Vitamin C (mg/d)	107 ± 108	221 ± 252*	138 ± 115	227 ± 121*	174 ± 120	273 ± 158*
Folate (μg/d)	211 ± 108	307 ± 140*	393 ± 138	500 ± 148*	423 ± 146	538 ± 166*
Potassium (g/d)	3.5± 1.2	3.8±1.7	3.6 ± 1.0	4.2 ± 1.1*	4.2 ± 1.2	4.2 ± 0.9

Data are expressed as means ± SD.

Statistical comparison (test t) was between non-consumers and consumers groups of normolipidemic women, normolipidemic men, and hypercholesterolemic men

*p < 0.05.

^#^ Normolipidemic: total cholesterol < 6.2 mmol/L.

^##^Hypercholesterolemic: total cholesterol ≥ 6.2 mmol/L.

Among women, 68% had a normal BMI and 32% exceeded the normal range. On the other hand, 42% of the men were normal weight and 58% were overweight and obese (80 and 20% respectively). These results suggested that a large percentage of the participants in this investigation were overweight; that is, 32% of the women and 58% of the men. Comparison of the anthropometric variables of all groups with normal and high cholesterol levels showed that regular orange juice consumption was not associated with body weight, BMI, waist circumference or percentage of body fat (p > 0.05) (Table 2). Hypercholesterolemia was present in 38% of the men, especially in those with excessive weight and increased waist circumference, while normocholesterolemic women and men showed an average BMI within normal range.

**Table 2:** Anthropometric and physical activity parameters of orange juice consumers and non-consumers according to gender and serum cholesterol level

	**Normolipidemic**				**Hypercholesterolemic**	
***Subjects***	**Women**		**Men**		**Men**	
n	18	8	38	26	23	16
Orange juice	Non-consumers	Consumers	Non-consumers	Consumers	Non-consumers	Consumers
mL/d	0	390 ± 149	0	466 ± 163	0	486 ± 145
Age (years)	37.6 ± 10.2	39.2 ± 12.2	36.8 ± 9.92	33.8 ± 10.9	41.9 ± 9.8	40.8 ± 8.0
Weight (kg)	64.0 ± 12.4	63.2 ±10.6	75.9 ± 11.4	70.8 ±10.0	78.5 ±13.8	83.0 ± 10.5
BMI (kg/m^2^)	24.5 ± 4.36	24.6 ±4.99	25.1 ± 3.63	23.8 ±2.89	26.6 ± 2.8	27.0 ± 3.4
Body fat (%)	36.7 ± 4.45	33.9 ± 6.53	27.8 ± 5.03	25.1 ± 7.52	28.0 ± 5.7	27.0 ± 4.1
Waist circ.(cm)	82.5 ±10.5	88.5 ± 12.3	90.4 ±9.96	87.5 ± 9.33	96.3 ± 8.2	97.5 ± 9.3
Physical Activity						
PA^###^	1.11 ± 0.11	1.16 ± 0.13	1.17 ± 0.11	1.15 ± 0.10	1.18 ± 0.08	1.17 ± 0.11
Duration (h/d)	0.66 ± 0.61	0.50 ± 0.44	1.18 ± 1.23	1.06 ± 0.79	0.89 ± 0.48	0.80 ± 0.64
Frequency (times/wk)	2.39 ± 2.59	3.38 ± 2.83	2.94 ± 2.37	2.75 ± 2.27	2.72 ± 1.74	3.23 ± 2.17
Sedentary^####^ (%)	56	38	16	15	5	29
Low Active (%)	11	38	37	46	50	31
Active (%)	33	25	45	38	45	40
Very Active (%)	0	0	3	0	0	0

Data are expressed as means ± SD.

Statistical comparison (test t) was between non-consumers and consumers groups of normolipidemic women and men, and hypercholesterolemic men.

*p < 0.05.

^#^ Normolipidemic: total cholesterol < 6.2 mmol/L.

^##^Hypercholesterolemic: total cholesterol ≥ 6.2 mmol/L.

^###^ PA is the physical activity coefficient according to DRI (2005).

^####^ % individuals with Sedentary, Low Active, Active or Very Active life style.

The comparison of physical activity levels between orange juice consumers and non-consumers (Table 2) showed no statistical differences (p > 0.05). In fact, the average physical activity coefficient (PA) ranged from 1.11 to 1.18, meaning low physical activity for both genders, among consumers and non-consumers, according to DRI-2005 [28]. The percentage of volunteers with sedentary or low active life style was higher (≥ 55%) than the active subjects in all groups (≤ 45%). Very active subjects were only detected among normolipidemic men, in very low proportion (3%).

Biochemical data of men and women with normal cholesterol levels and men with high cholesterol levels are presented in Table 3. Normolipidemic individuals who consumed orange juice regularly had lower total cholesterol (−11%), LDL-cholesterol (−18%), LDL/HDL ratio (−12%), and apo B (−12%) in comparison to normolipidemics without orange juice consumption (p ≤ 0.05). Similarly, hyperlipidemic men who consumed orange juice showed lower total cholesterol (−5%), LDL-cholesterol (−12%), apo B (−12%) and LDL/HDL ratio (−16%) in comparison to those without orange juice consumption (p ≤ 0.05) (Table 3). Although we did not investigate the family history of heart disease in this study, the identification of the hypercholesterolemic subjects showed the genetic heritage for heart disease in this group of people. On the other hand, triglycerides, HDL-cholesterol, apo A-I and homocysteine did not show any difference between consumers and non-consumers of orange juice (p > 0.05) (Table 3). All individuals had normal homocysteine levels (IMx Metabolic – Homocysteine, Abbott Laboratory).

**Table 3:** Biochemical characteristics of orange juice consumers and non-consumers according to gender and serum cholesterol level

	**Normolipidemic**		**Hypercholesterolemic**	
*n*	56	34	23	16
Orange juice	Non-consumers	Consumers	Non-consumers	Consumers
mL/d	0	530 ± 188	0	486 ± 145
Triacylglycerol (mmol/L)	1.15 ±0.37	1.11 ±0.55	1.86 ± 0.63	2.16 ±1.12
Cholesterol (mmol/L)				
Total	5.08 ±0.70	4.54 ±0.78*	6.77 ± 0.41	6.40 ±0.58*
HDL	1.20 ±0.29	1.16 ±0.31	1.14 ± 0.27	1.16 ± 0.23
LDL	3.35 ±0.61	2.74 ±0.80*	4.78 ± 0.48	4.20 ± 0.86*
LDL/HDL ratio	2.93±0.86	2.57 ±2.57*	4.46 ± 1.25	3.73 ±1.00*
Apolipoprotein (g/L)				
A-I	1.40 ±0.25	1.36 ±0.25	1.41 ± 0.28	1.39 ± 0.18
B	0.90 ±0.19	0.79 ±0.21*	1.27 ± 0.17	1.12 ± 0.17*
Homocysteine (μmol/L)	9.95 ±2.81	9.17 ±2.31	11.1 ±2.53	10.0 ± 2.31

Data are expressed as means ± SD.

Statistical comparison (test t) was between consumers and non-consumers groups of normolipidemic and hypercholesterolemic individuals.

*p < 0.05.

^#^ Normolipidemic: total cholesterol < 6.2 mmol/L.

^##^Hypercholesterolemic: total cholesterol ≥ 6.2 mmol/L.

Significant inverse correlations (data not showed) were found between regular orange juice intake and the following biochemical variables: total cholesterol (r = −0.35, p < 0.001), LDL-cholesterol (r = −0.38, p < 0.001), apo B (r = −0.30, p < 0.003) and LDL/HDL ratio (r = −0.24, p < 0.02) in men and women with normal cholesterol levels. Significant inverse correlations were also found between regular orange juice intake and total cholesterol (r = −0.31, p < 0.05), LDL-cholesterol (r = −0.39, p < 0.02) and apo B (r = −0.31, p < 0.05) in men with high cholesterol levels. An inverse correlation was found between men with regular orange juice intake and homocysteine levels (r = −0.20, p < 0.05), and homocysteine levels and apo A-I (r = −0.30, p < 0.001), while a positive correlation was found between homocysteine levels and triglycerides (r = 0.17, p < 0.04).

## Discussion

Recent studies have debated the nutritional risks and benefits of the regular consumption of fruit juices related to the increase of overweight and obesity [6,29-32]. There are some suggestions that calorie-free or low food energy content beverages (water, tea, coffee, low fat milk, skim milk, soy beverages) and non-calorically sweetened beverages, should be preferred to beverages with some nutritional benefits, such as fruit and vegetable juices, whole milk, alcoholic beverages and sports drinks, to prevent weight gain [29,31]. However, others have shown that consumption of pure fruit juices, without added sweeteners, did not contribute to weight gain [6,30,32], but protected against chronic diseases such as coronary heart disease and some cancers [33] because of the contribution of bioactive compounds, including flavonoids and vitamins. The statement that the consumption of orange juice contributes to the development of weight gain and obesity was not confirmed in the present study nor by a recent epidemiological study with children [34], since orange juice consumption was not associated with body weight, body fat, waist circumference, obesity or physical activity in this study. The majority of the consumers and non-consumers of orange juice showed low physical activity with no statistical difference among groups. So, we suggested that physical activity had no influence on the singularities detected on the markers of lipid metabolism or on the intake of vitamin C and folate, which were influenced by the consumption of orange juice.

Together with excess weight, visceral fat can promote metabolic disorders, such as insulin resistance, dyslipidemias and high blood pressure [22,35]. In our work, the percentage abdominal obesity among orange juice consumers did not differ from that of non-consumers. A meta-analysis showed that high consumption of fruits, juices and non-starchy vegetables may change body composition, reflected in BMI and abdominal obesity. Hence, consumption of these fresh foods may not help to reduce weight and waist circumference, but may help to delay or prevent their increase [36,37]. Also, orange juice has high nutritional density with low food energy content [38] in comparison with other unfortified juices and beverages, and helps consumers to meet the nutritional requirements of vitamin C, folate and potassium [39]. Orange juice can be considered a safe and protective energy source since it protects against oxidative or inflammatory stress [36].

Most participants of the present study followed a similar diet, since they had the two main meals of the day, breakfast and lunch, at the company’s restaurant from Monday through Friday. According to the dietary recalls, there was no difference among orange juice consumers and non-consumers regarding dietary energy, protein, carbohydrate, dietary fibers, total fruit, total fat, cholesterol, and potassium consumption. Moreover, the orange juice consumers showed significantly higher intake of vitamin C and folate, attributable to their average orange juice intake (480 mL/d). A previous experiment [18] verified a linear increase of vitamin C in the blood serum with increasing doses of orange juice, from 250 to 750 mL, while serum folate concentration only increased significantly when orange juice consumption reached 750 mL/d, suggesting that folate bioavailability is limited by its source and concentration in food.

Folate supplementation can decrease the homocysteine of individuals with originally normal and high levels, with dietary folate providing a beneficial impact on homocysteine levels [40]. Orange juice is one of the most common sources of folate in the human diet: 250 mL of orange juice contains 47 μg of folate [41], which may help to control serum homocysteine levels [18]. A mean orange juice intake of 480 mL/d provides an increase in dietary folate intake of 90 μg/d, and as much as 141 μg/d for those who consume 720 mL of orange juice. Most likely these increased folate intakes were responsible for the significant inverse correlation that was detected in the present study between orange juice and serum homocysteine levels, observed only for males.

The serum homocysteine levels of men with normal and high cholesterol levels who consumed orange juice daily did not differ significantly from those of their respective counterparts who did not consume orange juice daily. A previous study showed that consumption of orange juice below 750 mL/d did not affect serum homocysteine levels [18]. However, another author observed that serum homocysteine levels only decreased when daily folate intake reached 250 μg [42]. More recently, a study found that daily intake of 250 to 500 μg of folate reduced serum homocysteine levels by 11 to 20% [42]. Dosages below 250 μg/d do not seem to affect serum homocysteine levels significantly; nevertheless, orange juice still contributes to the total daily folate intake.

The present study found an inverse correlation between serum homocysteine levels and apo A-I, which is in agreement with a recent study of patients with coronary artery disease [42]. A positive correlation was also observed between serum homocysteine levels and serum triglycerides (r = 0.17, p < 0.04). A study in animals found a direct relationship between high homocysteine levels and changes in lipid metabolism that increased the risk of coronary artery disease [43]. Most participants, who consumed orange juice regularly, showed a slight reduction in homocysteine levels, that is, 8.5% in normolipidemic individuals and 11% in hypercholesterolemic individuals, although not significantly. High homocysteine levels in humans and animals are associated with endothelial dysfunction, accumulation of fats in the liver, low HDL- cholesterol and a slight change in total cholesterol [42,44]. An experimental study using supra physiological dosages of homocysteine found that the underlying mechanism involves inhibition of apo A-I synthesis in the liver and consequent reduction of serum HDL- cholesterol and its functional and anti-inflammatory activities on the endothelium [44].

The dietary intake of saturated fat and cholesterol of the normolipidemic orange juice consumers or non-consumers was close to the dietary guidelines of American Heart Association [23]. On the other hand, hypercholesterolemic subjects, both consumers and non-consumers, had higher intake of dietary cholesterol (> 300 mg/d), but the dietary saturated fat followed the recommended allowance (< 7% of total food energy intake). One of the strategies to reduce serum cholesterol according to the AHA is to consume foods containing stanol/sterol esters, such as orange juice fortified with stanol ester, or foods high in bioactive compounds which can improve the lipid profile [23].

The present study found that orange juice consumers had on average lower serum levels of total cholesterol and LDL-cholesterol in comparison with non-consumers. Presumably these effects are related to citrus flavonoid compounds found in orange juice [5,43]. In vivo studies have shown that naringenin and hesperitin inhibited ACAT and microsomal transfer protein (MTP) activities, which are responsible for the synthesis and esterification of the cholesterol in the liver [16], resulting in lower Apo B secretion and consequently lower LDL-cholesterol levels [45]. Also a significant negative correlation was found between orange juice consumption and total cholesterol, LDL-cholesterol and LDL/HDL ratio in humans [5,10,14,46]. These study results elucidate the beneficial effect of orange juice on serum LDL-cholesterol levels in agreement with in vivo studies that demonstrate an effective cholesterol-lowering action of the bioactive components of orange juice, such as flavanones and vitamin C [5,10,14,46].

Additionally a previous study showed a positive effect of orange juice on HDL-cholesterol levels with the consumption of 750 mL of orange juice per day [18], which was not verified in our study. Only 8% of our volunteers (data not shown) had a daily orange juice consumption that matched or exceeded the consumption of 750 mL per day, which may account for the difference.

The average of apo B and LDL-cholesterol of the orange juice consumers was lower in both normolipidemic and hyperlipidemic groups. Furthermore, an inverse correlation was found between regular orange juice consumption and apo B, reinforcing the LDL-cholesterol lowering effect of orange juice. An *in vitro* study found that hesperidin and naringin reduced the availability of lipids for assembly of apo B-containing lipoproteins: VLDL- and LDL-cholesterol, which is attributed to reduced ACAT 1 and ACAT 2 activities, selective decrease in ACAT 2 expression and reduced microsomal triglyceride transfer protein activity [16].

Serum triglyceride levels were statistically different between consumers and non-consumers of orange juice, despite the presence of sugars in the juice; however they were higher in the hypercholesterolemic group than in the normolipidemic. Animals given orange or grapefruit flavanones daily experienced a reduction in serum triglyceride levels [47] but studies with human beings have suggested that the magnitude of their effect on triglyceride levels depends primarily on other factors, such as age, gender, fasting glucose, insulin and triglyceride levels, insulin resistance and total fructose intake [36].

Although a smoking habit has been associated with a significant increase of stroke, high blood pressure, and damage to the arterial wall contributing to atherosclerosis, all of them considered as risk factors for cardiovascular disease [23], this behavior was not investigated in this study. The main focus of this investigation were the markers of lipid metabolism due to the benefit of the orange juice on total cholesterol and LDL-cholesterol in women and hyperlipidemic men, as previously detected from these authors [13,14].

The first strength of this study was the volunteers have been already exposed to daily orange juice consumption and that they freely chose to consume or not. The second strength of this study was the exposure of individuals to the same main meals every day (breakfast and lunch). This environment was more homogeneous than individuals who had their meals at home or in different places. Nevertheless, be exposed to the same food environment, does not mean they ate the same foods. Therefore, the assessment of food consumption was conducted through the individual 24-hour recall. The limitation, however, was that the dietary information was obtained through a self-reported questionnaire, which may be subject to under or over reporting and interviewer bias, and because of the cross-sectional design, which is limited in verifying any relationship between cause and effect.

## Conclusions

This study suggested that long-term consumption of orange juice by normolipidemic and hypercholesterolemic subjects was not associated with weight or body fat, or plasma triglyceride and homocysteine. It was, however, associated with reduced total cholesterol, LDL-cholesterol, apo B and LDL/HDL ratio. Hence, it corroborated the hypothesis that long-term orange juice consumption was significantly associated with favorable effects on risk factors for cardiovascular disease, suggesting that future research should test whether orange juice can be useful as a secondary intervention in subjects with normal or moderately high serum cholesterol.

## Methods

### Subjects and study design

It was a descriptive and cross-sectional study with healthy volunteers who worked at *Citrosuco* (Fischer S/A – Comercio Industria e Agricultura), an orange juice factory located in Matao, SP, Brazil. A total of 129 volunteers were selected to participate in this study, 103 men aged 18 to 66 y and 26 women aged 18 to 65 y. None of the 129 participants had thyroid, kidney, or heart disease, diabetes, or required the use of pharmaceuticals, and those who consumed more than zero but less than one cup of orange juice per day (>0 or <240 mL/d) were excluded of the sample as well. The study was approved by the Ethics Committee of the School of Pharmaceutical Sciences, São Paulo State University, and an informed written consent was obtained from each participant.

General and anthropometric data were collected over a four-week period by the researchers of this study. During the first week of the experiment, blood samples were obtained from the median cubical vein and placed in EDTA-containing vials. Serum samples were centrifuged at 3,000 × g for 10 min at room temperature within 1 h of collection and stored at −80°C until the assays of total cholesterol, HDL-cholesterol, triglycerides, apoA1, apo B, homocysteine, were performed at the Clinical Analyses Laboratory, School of Pharmaceutical Sciences, UNESP, Araraquara, SP. Reagents and instruments used for these analyses are described on the Biochemical Tests section.

Daily consumption of foods and beverages was determined with two 24-h recalls, described in “Dietary assessment” section, conducted by the researchers during four weeks of the experimental period. Nutritional requirements were estimated according to the National Research Council [28]. The frequency of the consumption of orange juice was obtained with the first dietary recall and all consumers stated that their primary source was the orange juice from the company cafeteria. The specific question addressed to the volunteers was: “How much and how often did you drink orange juice during the last 12 months?” Those who reported drinking orange juice every day, one to more cups per day, in the last twelve months were selected as habitual consumers of orange juice (n = 50), with an average intake of 480 ± 158 mL/d. Those who did not drink orange juice during the last twelve months (0 cup/d), as non-consumers (n = 79). Those who consumed some orange juice, but less than one cup per day (240 mL/d) were excluded of the sample. All collected data were observational and descriptive of the usual and spontaneous orange juice intake by the volunteers.

Dietary, physical activity and anthropometric variables of men and women were analyzed separately because of the distinct gender characteristics for these variables. Because dyslipidemias are under strong genetic influence, the volunteers were divided into those with normal cholesterol levels (n = 90, total cholesterol < 6.2 mmol/L) and those with moderately high cholesterol levels (n = 39, total cholesterol ≥ 6.2 mmol/L) in order to evaluate the individual influence of orange juice on the blood lipid profile.

### Orange juice composition

Commercially processed orange juice (12° Brix, reconstituted from concentrate) was provided daily in the company restaurant as their primary source of orange juice as reported by the volunteers. The average daily serving of orange juice (480 mL) contained approximately 161 mg of vitamin C, 90 μg of folate, 851 mg of potassium, 40 g of total sugar (19 g of sucrose, 10 g of glucose, 11 g of fructose) and 970 kJ (234 kcal) [48]. The flavonoid content in 480 mL of orange juice was estimated at 26.4 mg of hesperitin and 7.4 mg of naringenin [48]. Daily orange juice consumption estimates were determined indirectly by self-reporting from all the volunteers.

### Dietary assessment

Daily consumption of food and beverage was determined by a 24-hour food recall and analyzed with a computer-based data evaluation system (NUTRI software, version 2.5 CIS, Federal University of Sao Paulo School of Medicine, Brazil). Food intakes were described in terms of portion sizes, and sugar and lipid consumption was estimated from the food preparation. Food energy intake was estimated from the consumption of macronutrients in the 24-hour food recall (Table 1), where 1 g of protein or carbohydrate equals 16.7 kJ and 1 g of lipid equals 37.7 kJ. The nutritional requirements of all individuals were estimated according to the National Research Council [28].

### Anthropometric and physical activity assessment

Body weight (kg) and height (m) were measured for all volunteers and body mass index (BMI) was determined (weight/height^2^) [49]. Waist circumference (narrowest diameter between xiphoid process and iliac crest) was measured in triplicate at one time for each subject, and the average was taken as the final determination [50]. Bipolar body electrical bioimpedance was assessed early in the morning to determine body fat, before the participants had broken the overnight fast (Biodynamics Model 310e, Seattle, WA, USA). An electric current of 0.8 mA and 50 kHz was produced by a calibrated signal generator and applied to the skin using adhesive electrodes placed on right-side limbs [50].

Physical activity was investigated with the first 24-h recall, by four specific questions: practice or non-practice of physical activity, type of exercise, duration (h/d) and frequency (times/wk). All these parameters were used to determinate the physical activity coefficient (PA), which represents four categories of life style, as sedentary, low active, active and very active, according to gender and BMI [28].

### Biochemical tests

Total serum cholesterol (CHOD-PAP; Roche), triacylglycerol (Triglyceride Rapid; Roche), and HDL-cholesterol (HDL Reagent Roche) were determined enzymatically by an automated biochemical analyzer (Roche Cobas e601, Switzerland). LDL-cholesterol was estimated by Friedewald’s equation (1972) [51]. Apolipoprotein A (apo A-I) and apolipoprotein B (apo B) were determined by immunoturbidimetric assay using commercial kits (Roche Diagnostics, Germany) in a Cobas MIRA analyzer (Roche, Switzerland). Homocysteine (IMx Metabolic - Homocysteine, Abbott Laboratory) was determined by the Axsym system immunological method by fluorescence polarization (AXSYM, Abbott, Wiesbaden, Germany).

### Statistical analyses

Sample size was calculated to detect means differences with 90% power and 5% significance. Data were expressed as mean ± standard deviation. The distribution of variables was analyzed with Kolmogorov-Smirnov test. Variables with normal distribution were analyzed with a Student’s *t*-test. Non-parametric variables were analyzed with the W-Wilcoxon test. Associations between orange juice consumption and variables were analyzed using a Spearman’s correlation. p-value of 0.05 was used to define significance. The data were analyzed using Sigma Stat for Windows® (version 3.11, 2004 Systat Software Inc, USA).

## Abbreviations

ACAT acyl CoA: Cholesterol acyltransferase; apo A-I: Apolipoprotein A; apo B: Apolipoprotein B; BMI: Body mass index; HDL: High-density lipoprotein; HMG-CoA 3: Hydroxy-3-methyl-glutaryl coenzyme A; LDL: Low-density lipoprotein; MTP: Microsomal transfer protein; VLDL: Very low-density lipoprotein.

## Competing interests

The authors state that there is no conflict of interest and that they are responsible for the content and writing of the paper.

## Authors’ contributions

NPA has collected and arranged the data of the trial and TBC drafted the manuscript. TBC was the principal investigator of the project and has made the conception and design of the study, and also guided in the analysis and statistical design of research trial. NPA helped to carry out the statistical analysis. Both authors have given final approval of the version to be published.
